# Simulated Medicare Drug Price Negotiation Under the Inflation Reduction Act of 2022

**DOI:** 10.1001/jamahealthforum.2022.5218

**Published:** 2023-01-27

**Authors:** Benjamin N. Rome, Sarosh Nagar, Alexander C. Egilman, Junyi Wang, William B. Feldman, Aaron S. Kesselheim

**Affiliations:** 1 Program on Regulation, Therapeutics, and Law (PORTAL), Division of Pharmacoepidemiology and Pharmacoeconomics, Department of Medicine, Brigham and Women’s Hospital, Boston, Massachusetts; 2 Harvard Medical School, Boston, Massachusetts; 3 Division of Pulmonary and Critical Care Medicine, Department of Medicine, Brigham and Women’s Hospital, Boston, Massachusetts

## Abstract

**Importance:**

The Inflation Reduction Act of 2022 gives Medicare the authority to negotiate prices for certain prescription drugs. Which drugs will be selected and how prices will be negotiated remain unclear.

**Objective:**

To simulate drug selection and the minimum savings that would have been achieved at statutory ceiling prices if Medicare drug price negotiation had been implemented from 2018 to 2020.

**Design, Setting, and Participants:**

In this cross-sectional study, a policy simulation analysis of high-spending prescription drugs in Medicare Part B and Part D that were eligible for negotiation from January 2018 to December 2020 was performed from August 5 to November 20, 2022.

**Exposures:**

Eligibility criteria for selection and discounts afforded by the statutory ceiling prices for negotiation.

**Main Outcomes and Measures:**

The main outcomes were characteristics of drugs subject to negotiation and estimated Medicare savings from 2018 to 2020 that would have been achieved through spending at ceiling prices compared with existing net prices accounting for price concessions.

**Results:**

Among the 40 selected drugs, 35 were primarily reimbursed through Medicare Part D and 5 through Part B and 10 were biologics. The most common therapeutic classes were endocrine (11), neurologic or psychiatric (5), pulmonary (4), rheumatologic or immunologic (4), and cardiovascular (4). Median time from US Food and Drug Administration approval to selection was 12 years (IQR, 10-14 years). Three drugs faced generic competition in the 2 years between selection and price negotiation. For the remaining 37 drugs, estimated net Medicare spending from 2018 to 2020 was $55.3 billion; spending at ceiling prices would have been reduced by an estimated $26.5 billion, which represented 5% of estimated net Medicare drug spending during those 3 years.

**Conclusions and Relevance:**

In this cross-sectional study, simulating the drug price negotiation provisions in the Inflation Reduction Act of 2022 revealed important limitations, including strict selection criteria and the potential for drugs to become ineligible for negotiation during the 2 years between selection and prices taking effect. Despite these limitations, the policy still delivered substantial savings because ceiling prices offered steep discounts, in part, by erasing excess spending from price increases faster than inflation.

## Key Points

**Question:**

How much would Medicare price negotiation under the Inflation Reduction Act of 2022 have reduced prescription drug spending if the policy had taken effect from 2018 to 2020?

**Findings:**

In this cross-sectional study using a policy simulation analysis, 40 top-selling drugs that would have been selected for negotiation from 2018 to 2020 were identified; although individual Medicare Part D plans already received substantial discounts for many of these drugs, the statutory ceiling prices for negotiation would have reduced spending by $26.5 billion on these drugs, or 5% of estimated net Medicare drug spending, from 2018 to 2020.

**Meaning:**

The findings suggest that despite restrictive selection criteria for negotiation, the Inflation Reduction Act of 2022 will likely result in substantial savings in the first 3 years for many high-spending drugs.

## Teaser

This cross-sectional study uses a policy simulation analysis to assess how much Medicare price negotiation would have reduced prescription drug spending if the Inflation Reduction Act of 2022 had been effective from 2018 to 2020.

## Introduction

The Inflation Reduction Act of 2022 (IRA) included 3 major Medicare reforms aimed at lowering prescription drug costs: Medicare Part D benefit redesign, including a $2000 annual limit on out-of-pocket costs; mandatory rebates that penalize drug manufacturers for raising prices faster than inflation; and price negotiation for a set of high-cost drugs.^1,2^ While all 3 policies will improve prescription drug affordability in the US, the price negotiation provisions are expected to yield the largest savings for the federal government.^3^

Medicare is the single largest purchaser of prescription drugs in the US,^4^ and those drugs account for more than 1 in 4 health care dollars spent by Medicare.^5^ However, prior to passage of the IRA, Medicare was prohibited from negotiating prices.^6^ The private companies that operate Medicare Part D plans and their pharmacy-benefit managers have historically negotiated rebates and other discounts, typically in exchange for favorable formulary placement or for waiving utilization management tools (eg, prior authorization). Yet, even after accounting for these discounts, the average price paid by Medicare for brand-name drugs more than doubled from 2009 to 2018.^7^

The IRA limits the Centers for Medicare & Medicaid Services (CMS) to negotiating up to 20 high-spending drugs each year, which can only qualify after being on the market for at least 9 years (13 years for biologic products). Even with these restrictions, the Congressional Budget Office (CBO) estimated that price negotiation could save the federal government $100 billion from 2022 through 2031.^3^ However, the CBO’s published methods include assumptions about the negotiation process, and actual savings will depend on how the policy is implemented.^8^

Many questions remain about the implementation of CMS’s new negotiation authority, including which drugs will be selected and how the process will run. To evaluate the potential impact of this policy, we simulated drug selection and estimated savings if the IRA had taken effect from 2018 to 2020. Unlike the CBO’s estimates, historical simulation is not intended to project future savings. Instead, this analysis provides insight into several elements of the negotiation policy to help inform implementation by CMS.

## Methods

In this cross-sectional study, we modeled the selection of drugs and prices for the Medicare negotiation process based on statutory criteria in the IRA using the 3 most recent years of Medicare spending data that were available (January 2018 to December 2020). This study was not submitted for institutional review board approval because it used public, nonidentifiable data and did not constitute human participant research (45 CFR §46.102). The study was reported in accordance with the Strengthening the Reporting of Observational Studies in Epidemiology (STROBE) reporting guideline. Analyses were performed from August 5 to November 20, 2022.

### IRA Drug Selection

Under the IRA, CMS will select eligible drugs each year for negotiation; negotiated prices take effect 2 years after drugs are selected. The law specifies selection of 10 drugs in the first year, 15 drugs in the second and third years, and 20 drugs in each subsequent year, and CMS must select eligible drugs with the highest gross Medicare spending in the year prior to selection. In the first 2 years of the program, Medicare Part D (retail pharmacy) drugs will be considered; thereafter, Part B (clinician-administered) drugs will also be included. In selecting drugs, CMS must aggregate spending data across dosage forms and strengths, “including new formulations of the drug, such as extended-release formulations.”^1^

Eligible drugs must have annual Medicare spending exceeding $200 million, must have been approved by the US Food and Drug Administration (FDA) for at least 7 years (or 11 years for biologics), may not have any marketed generic or biosimilar competitors, cannot be approved to treat a single rare condition (as designated under the Orphan Drug Act), and cannot be a plasma-derived product (eg, intravenous immunoglobulins). For the first 3 years of the program, drugs cannot be selected if they represent less than 1% of Medicare Part B or Part D spending and are made by a company that received more than 80% of its Medicare revenue from a single, an exclusion designed to protect small biotechnology firms. For biologic products, either the originator or biosimilar manufacturer can delay selection if they attest that a biosimilar competitor will be marketed within 2 years, with financial penalties if a biosimilar does not enter the market during this time.

### Simulated Drug Selection

**Figure 1.**
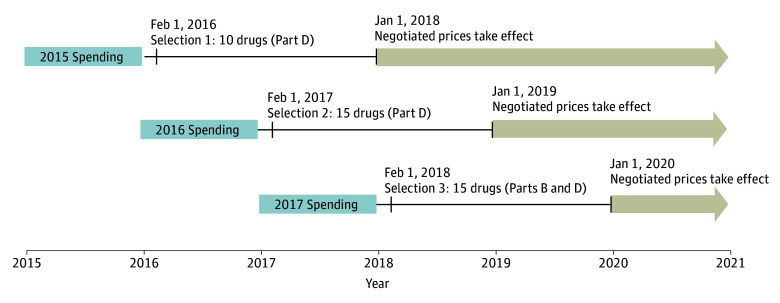
Timeline of Simulated Drug Selection and Negotiated Prices We simulated 3 years of drug selection based on the timeline specified in the Inflation Reduction Act of 2022 (IRA); drugs will be selected on February 1 of each year, with negotiated prices taking effect 2 years later. We did not account for a slightly different timeline specified by the IRA for the first year of the program; the first drugs will be selected on September 1, 2023, with negotiated prices taking effect in 2026. Selection will be based on drugs with the highest Medicare spending in the previous year. We simulated this by assuming drugs would be selected in 2016, 2017, and 2018, based on those with the highest Medicare spending in 2015, 2016, and 2017. Simulated negotiated prices took effect in 2018, 2019, and 2020 and lasted until a drug faced generic or biosimilar competition or through the end of our study on December 31, 2020.

We designed the simulation such that negotiated prices for selected drugs would take effect in 2018, 2019, and 2020 (Figure 1). We used publicly available annual Medicare Part D data from 2015 and 2016 (to simulate selection in 2016 and 2017) and Medicare Part B and Part D data from 2017 (to simulate selection in 2018).^9^ The published Medicare Part D spending data are partially aggregated; we further combined spending on multiple dosage forms of some drugs with the same active ingredient, such as pens and vials. We determined eligibility based on the IRA criteria, including the small biotechnology firm exclusion and excluding biologic drugs if a biosimilar version was marketed within the 2 years following the selection date. We used the date of approval for each drug from public FDA documents.^10^ We determined when biosimilar or generic versions were first marketed using public Medicaid data.^11,12^ We compared Orphan Drug Act designations in the FDA’s database^13^ with drug labeling to identify products approved to treat a single rare disease.

### Ceiling Prices Under the IRA

The law instructs CMS to negotiate a maximum fair price to take effect 2 years after a drug is selected; negotiated prices remain in effect until the calendar year that begins at least 9 months after the first generic or biosimilar version is marketed. The negotiated maximum fair price must fall below a ceiling price, which is the lesser of (1) the average net price after existing plan-negotiated rebates and discounts or (2) a percentage of the drug’s nonfederal average manufacturer price (non-FAMP): 75% for drugs that were approved less than 12 years ago, 65% for drugs that were approved 12 to 16 years ago, and 40% for drugs that were approved 16 or more years ago. The middle 65% category will not be included until 2030; thus, for the first 5 years, the ceiling price will be 75% of non-FAMP for drugs approved less than 16 years ago. If a drug’s price increased faster than inflation, the ceiling price is based on the non-FAMP from 2021 (or from the year after launch), adjusted for inflationary changes through the year prior to selection (eMethods in Supplement 1).

### Simulated Ceiling Prices

For each selected drug, we calculated the discounts from reported Medicare spending that would be necessary to achieve the ceiling price in each year that the drug was eligible for negotiation. Because non-FAMP data are confidential,^14^ we used the wholesale acquisition cost (WAC; ie, list price) obtained from AnalySource (with permission from First Databank). We adjusted WAC prices to estimated non-FAMP based on a recent CBO report finding that the average non-FAMP was 18% lower than the average WAC among top-selling drugs in 2017.^14^

In accordance with the IRA process, we based the ceiling price on the lower of the drug’s non-FAMP in the year prior to selection or the non-FAMP from 2013 (5 years before the first negotiated drug price would have taken effect in 2018), adjusted for inflationary changes using the Consumer Price Index for all urban consumers.^15^ Based on each drug’s time since FDA approval, at the time of selection, we estimated the ceiling price to be 75% (<16 years) or 40% (≥16 years) of the estimated non-FAMP. We determined discounts by comparing each drug’s estimated ceiling price with the retail price (assumed to be 7% lower than WAC^14^) in the years that the drug was eligible for negotiation (2018-2020).

### Statistical Analysis

We simulated savings from the ceiling prices for selected drugs by comparing the drug’s existing estimated discounts (ie, negotiated rebates) with the ceiling price discount (eMethods in Supplement 1). We assumed that use of these medications would remain unchanged even if prices were lowered by negotiation—an assumption we believed was reasonable because selected drugs are already top sellers in Medicare.

For Part B drugs, we estimated existing discounts from the list price by comparing the WAC with the publicly available Medicare payment limit (based on the average postrebate price paid by private insurers).^16^ For Part D drugs, we estimated confidential rebates based on annual average non-Medicaid rebate estimates from SSR Health, LLC (eMethods in Supplement 1).^17,18^ Analyses were performed using Excel, version 2202 (Microsoft Corp).

## Results

We simulated that Medicare would have selected 10 of the 14 drugs with the highest Part D spending in the first year, 15 of the 45 drugs with the highest Part D spending in the second year, and 15 of the 87 drugs with the highest combined Part B and Part D spending in the third year (Table 1). Most drugs were excluded because they were approved recently (51 drugs), already faced generic or biosimilar competition (14 drugs), or had been selected in a previous year (33 drugs). One plasma product (immune globulins [Gammagard]) was excluded. One drug approved to treat a single rare disease (glatiramer, which has an Orphan Drug Act designation for treating multiple sclerosis) was excluded in 2017 and 2018. In 2018, 3 biologics (bevacizumab, pegfilgrastim, and trastuzumab) were excluded because they faced biosimilar competition in the subsequent 2 years, and 2 products were excluded based on the rule protecting small biotechnology firms: extended-release oxycodone (Oxycontin) and lurasidone. For a list of all exclusions, see eTable 1 in Supplement 1.

**Table 1.** Reasons Drugs Were Excluded From Selection, 2016-2018

	Selection date		
	February 1, 2016 (n = 14)	February 1, 2017 (n = 45)	February 1, 2018 (n = 87)
Selected, No. (%)	10 (71)	15 (33)	15 (17)
Excluded, No. (%)	4 (29)	30 (67)	72 (83)
Treats a rare disease^a^	NA	1 (3)	1 (1)
Plasma product^b^	NA	NA	1 (1)
Insufficient time since approval^c^	2 (50)	15 (50)	34 (47)
Generic or biosimilar competition^d^	2 (50)	4 (13)	8 (11)
Biosimilar within 2 y^e^	0	0	3 (4)
Small biotechnology firm exclusion	0	0	2 (3)
Previously selected	0	10 (33)	23 (32)

^a^ Drug was approved by the US Food and Drug Administration to treat a single disease with Orphan Drug Act designation. Only glatiramer met this criterion, which led to its exclusion in 2017 and 2018.

^b^ The only top-selling plasma product excluded from selection was immune globulins (Gammagard).

^c^ Approved for less than 7 years for small-molecule drugs and less than 11 years for biologics.

^d^ A generic or biosimilar version was marketed prior to the selection date.

^e^ A biosimilar version was marketed within 2 years after the selection date. In these cases, we assumed the manufacturer would have successfully requested delayed selection.

Among the 40 selected drugs, 35 were primarily reimbursed under Medicare Part D and 5 under Part B. Ten drugs were biologics, and all drugs had Medicare spending exceeding $400 million in the year prior to selection (Table 2). The most common therapeutic classes included endocrine (11, including 5 insulins), neurologic or psychiatric (5), pulmonary (5), rheumatologic or immunologic (4), and cardiovascular (4) drugs. Drugs were selected a median of 12 years (IQR, 10-14 years) after FDA approval; the median time from approval to selection was 15 years (IQR, 12-16 years) for biologics and 12 years (IQR, 9-13 years) for small-molecule drugs.

**Table 2.** Simulated Drugs Selected for Medicare Price Negotiation, 2016-2018

Selected drug by year	Medicare spending, million $^a^	Drug type	Time since FDA approval, y	Date of first generic or biosimilar competition^b^
2016 (Medicare Part D)				
Insulin glargine (Lantus)	4340	Biologic^c^	15.8	August 2020
Rosuvastatin (Crestor)	2883	Small molecule	12.5	May 2016^d^
Fluticasone/salmeterol (Advair)	2432	Small molecule	15.4	February 2019
Tiotropium (Spiriva)	2302	Small molecule	12.0	NA
Sitagliptin (Januvia)	2132	Small molecule	9.3	NA
Lenalidomide (Revlimid)	2077	Small molecule	10.1	NA
Pregabalin (Lyrica)	1766	Small molecule	11.1	July 2019
Insulin aspart (Novolog)	1686	Biologic	15.7	NA
Adalimumab (Humira)	1662	Biologic	14.1	NA
Memantine (Namenda)	1628	Small molecule	13.3	February 2018
2017 (Medicare Part D)				
Insulin detemir (Levemir)	1950	Biologic^c^	11.6	NA
Insulin lispro (Humalog)	1803	Biologic^c^	20.6	NA
Etanercept (Enbrel)	1572	Biologic	18.3	NA
Sevelamer (Renvela)	1462	Small molecule	9.3	June 2017^d^
Budesonide/formoterol (Symbicort)	1432	Small molecule	10.5	NA
Cinacalcet (Sensipar)	1226	Small molecule	12.9	December 2018
Liraglutide (Victoza)	1003	Small molecule	7.0	NA
Cyclosporine eye drops (Restasis)	949	Small molecule	13.3	NA
Paliperidone (Invega Sustenna)	917	Small molecule	7.5	NA
Sitagliptin/metformin (Janumet)	729	Small molecule	9.8	NA
Solifenacin (Vesicare)	706	Small molecule	12.2	April 2019
Emtricitabine/tenofovir disoproxil fumarate (Truvada)	636	Small molecule	12.5	September 2020
Ambrisentan (Letairis)	574	Small molecule	9.6	April 2019
Dexlansoprazole (Dexilant)	550	Small molecule	8.0	NA
Efavirenz/emtricitabine/tenofovir disoproxil fumarate (Atripla)	533	Small molecule	10.6	October 2020
2018 (Medicare Parts B and D)				
Ranibizumab (Lucentis)^e^	1041	Biologic	11.6	NA
Abatacept (Orencia)^e^	929	Biologic	12.1	NA
Corticotropin (Acthar)	681	Small molecule	7.3	NA
Dabigatran (Pradaxa)	646	Small molecule	7.3	NA
Insulin aspart mix (Novolog Mix 70/30)	631	Biologic	16.3	NA
Ranolazine (Ranexa)	599	Small molecule	12.0	February 2019^d^
Teriparatide (Forteo)	576	Small molecule	15.2	NA
Rifaximin (Xifaxan)	541	Small molecule	13.7	NA
Omalizumab (Xolair)^e^	500	Biologic	14.6	NA
Pemetrexed (Altima)^e^	483	Small molecule	14.0	NA
Octreotide (Sandostatin LAR)^e^	471	Small molecule	19.2	NA
Albuterol (Ventolin)	464	Small molecule	16.8	NA
Lacosamide (Vimpat)	462	Small molecule	9.3	NA
Fingolimod (Geilenya)	457	Small molecule	7.4	NA
Albuterol (ProAir)	406	Small molecule	13.3	August 2020

Abbreviations: FDA, US Food and Drug Administration; NA, not applicable.

^a^ Spending was in the full calendar year prior to selection. In 2016 and 2017, only gross Medicare Part D spending was included. In 2018, Medicare Part B spending and gross Medicare Part D spending were combined.

^b^ Through December 2020.

^c^ Insulins were changed from being regulated as small molecules to biologics in 2020. Despite simulating selection before 2020, insulins were treated as biologics.

^d^ Generic competition occurred after simulated drug selection but before the negotiated price would have taken effect 2 years later.

^e^ Selected from the top-selling drugs in Medicare Part B. All other drugs were top-selling drugs in Medicare Part D.

Three of the 40 selected drugs faced generic competition within the subsequent 2 years after selection: ranolazine, rosuvastatin, and sevelamer. The remaining 37 drugs were eligible to face negotiated prices for at least 1 year from 2018 to 2020. Three drugs selected in 2015 or 2016 faced generic competition before 2020 and thus became ineligible for a negotiated price: cinacalcet (became ineligible in 2020), fluticasone/salmeterol (became ineligible in 2020), and memantine (became ineligible in 2019). Overall, 9 selected drugs were eligible for negotiated prices in 2018, 22 drugs in 2019, and 34 drugs in 2020 (Table 3).

**Table 3.** Medicare Spending and Savings From Drugs Selected for Negotiation, 2018-2020

	Year			2018-2020
	2018	2019	2020	
Medicare spending for all drugs, billion $^a^				
Part D, prerebate	168.1	183.0	198.7	549.8
Part D, estimated net	119.2	124.4	131.6	375.2
Part B^b^	33.3	37.3	38.5	109.1
Total (Part B and net Part D)	152.5	161.7	170.1	484.3
Selected drugs eligible for negotiated prices, No.^c^	9	22	34	37
2016	9	8	7	9
2017	NA	14	13	14
2018	NA	NA	14	14
Medicare spending for selected drugs, billion $ (% of Medicare drug spending)				
Part D, prerebate	24.3 (14)	39.9 (22)	41.7 (21)	106.0 (19)
Part D, estimated net	13.0 (11)	18.7 (15)	20.1 (15)	51.8 (14)
Part B^b^	NA	NA	3.5 (9)	3.5 (3)
Total (Part B and net Part D)	13.0 (9)	18.7 (12)	23.6 (14)	55.3 (11)
Discount to achieve ceiling prices, median (IQR), %^d^	70 (60-77)	66 (61-75)	66 (58-77)	66 (58-77)
Selected drugs with ceiling price discounts exceeding existing discounts, No. (%)^e^	7 (78)	14 (61)	25 (74)	29 (78)
Savings from ceiling prices, billion $ (% of Medicare drug spending)				
Part D	5.9 (5)	8.6 (7)	10.3 (8)	24.8 (7)
Part B	NA	NA	1.7 (4)	1.7 (2)
Total	5.9 (4)	8.6 (5)	12.0 (7)	26.5 (5)

Abbreviation: NA, not applicable.

^a^ Prerebate Medicare Part D spending and Part B spending were obtained from public Medicare annual spending reports (ie, Centers for Medicare & Medicaid Services Drug Spending Dashboards). Estimated net Medicare Part D spending after rebates and discounts was calculated using the method reported by Feldman et al.^19^

^b^ Medicare Part B spending was based on the average sales price, which included average discounts to some private payers.

^c^ After drugs were selected, negotiated prices were effective beginning 2 years after selection until the first year starting 9 months after a generic or biosimilar version was marketed. For example, memantine was selected in 2016, so a negotiated price took effect in 2018. However, the drug faced generic competition beginning in February 2018, so the negotiated price was not effective for 2019 or 2020.

^d^ Percentage reduction in Medicare prerebate spending from the ceiling price, calculated based on the difference between the estimated ceiling price and the drug’s price from 2018 to 2020.

^e^ Existing discounts included estimated confidential rebates received by Medicare Part D plans. For drugs with ceiling price discounts less than existing discounts, the average net price after rebates became the ceiling price.

### Ceiling Prices

The median ceiling price was 66% (IQR, 60%-77%) lower than retail Medicare spending. Twenty-five of the 37 selected drugs (68%) were approved in 2004 or later; thus, in most cases, ceiling prices were set at 75% of non-FAMP. However, prices for most drugs increased faster than inflation after 2013, resulting in a larger difference between the estimated ceiling price and the drug’s retail price from 2018 to 2020. For example, each of the 9 drugs selected in 2016 had price increases of at least 15% from 2013 to 2015 (median, 34%; IQR, 21%-46%), while inflation only increased by 2%.

For 29 of the 37 drugs (78%), the ceiling prices offered greater discounts than existing rebates. For the remaining 8 drugs (22%), existing rebates were larger than the ceiling price discounts, so simulated savings were 0 (ie, Medicare would only realize savings if it were able to negotiate lower prices than the ceiling price).

### Estimated Savings

**Figure 2.**
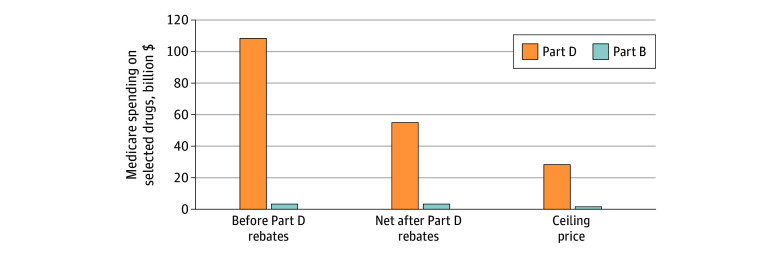
Estimated Medicare Spending on Drugs Eligible for Negotiation, 2018-2020 Medicare spent an estimated $55.3 billion on the 37 selected drugs from 2018 to 2020, after subtracting $54.2 billion in rebates negotiated by Part D plans. Spending would have been reduced by $26.5 billion (48%) if Medicare had paid the ceiling prices for negotiation, with most of the savings in Medicare Part D.

Combined estimated net Part B and Part D spending for the 37 drugs eligible for negotiated prices was $55.3 billion from 2018 to 2020 after deducting $54.2 billion (49%) that was offset by existing Part D rebates (Figure 2). This represented 11% of the estimated $484.3 billion Medicare spending on all prescription drugs from 2018 to 2020 (Table 3).^19^

If CMS had set maximum fair prices at the statutory ceiling, Medicare would have saved $26.5 billion ($1.7 billion in Part B and $24.8 billion in Part D). This would have lowered net spending on negotiated drugs by 48% and would have lowered total Medicare drug spending by 5%. Savings increased from $5.9 billion (4% of Medicare drug spending) in 2018 to $8.6 billion (5%) in 2019 and $12.0 billion (7%) in 2020.

The drug with the largest estimated savings from ceiling prices was lenalidomide, which cost Medicare $14.1 billion from 2018 to 2020 and would have cost only $5.2 billion at the ceiling price, yielding savings of $8.9 billion (63%). Other drugs with high estimated savings were adalimumab ($5.9 billion [74%]), pregabalin ($1.9 billion [55%]), long-acting paliperidone ($1.8 billion [56%]), and etanercept ($1.4 billion [70%]). Estimated annual savings for each of the 37 drugs are shown in eTable 2 in Supplement 1.

## Discussion

The IRA will allow Medicare to start directly negotiating prices for a limited set of drugs. Our simulation showed that ceiling prices for negotiation may lead to a substantial reduction in Medicare drug spending within the first few years. If the law had been implemented from 2018 to 2020, we estimated that Medicare prescription drug spending would have been reduced by $26.5 billion, or 5% of total Part B and Part D drug spending during that 3-year period.

The estimated savings are based on historical data and may not predict future savings. The CBO projected savings of $33 billion in first 3 years of negotiation (2026-2028) compared with $26.5 billion in our model.^3^ The assumptions underpinning the CBO’s estimates are unknown, but in a previous model, it assumed Medicare would negotiate value-based prices using reports from the Institute for Clinical and Economic Review.^8^ Our estimates suggest that the ceiling prices will likely result in substantial savings, but actual savings could be higher if CMS seeks prices below the ceiling.

Our simulation analysis highlights several important components of implementation of the IRA. First, the IRA only allows price negotiation for drugs that have been available for 9 years (13 years for biologics). We found that this limitation would have prevented selection of 34 high-spending drugs in 2018. By contrast, other industrialized countries negotiate prices for new drugs soon after launch.^20,21^ Delayed negotiation is particularly impactful because launch prices for new drugs increased by 11% per year from 2008 to 2021.^22^ Additional legislative or executive solutions may be needed to address this trend.^2^

Second, the IRA provides a rigid drug-selection process, instructing CMS to select drugs in order of highest spending. In a prior version of the legislation, CMS would have been allowed to choose among the 50 highest-spending eligible drugs,^1^ but this flexibility was dropped from the final law. In addition, drugs are selected 2 years before negotiated prices take effect; while the list of top-selling drugs is similar from year to year, some changes could have consequences. For example, the IRA allows biologic manufacturers to avoid selection if biosimilar competition is anticipated within 2 years, but there is no provision that allows CMS to avoid selecting small-molecule drugs with impending generic competition. We identified 3 cases in which top-selling drugs faced generic competition within 2 years after selection, before negotiated prices would have taken effect. Because Medicare can only select a limited number of drugs each year, choosing these drugs would lead to missed opportunities to address high prices of other drugs.

The selection process may lead CMS to negotiate prices for 1 drug within a class but not others, which could have unanticipated market effects. For example, sitagliptin would have been selected in 2016, while other dipeptidyl peptidase-4 inhibitors did not have high enough Medicare spending to qualify for selection. For Medicare Part B drugs, reimbursement is set based on a fee schedule, so this could lead to variable prices for drugs in the same therapeutic class. For Medicare Part D drugs, negotiating 1 drug could place pressure on competitors to match the government-negotiated prices. However, this might be accomplished through rebates to maintain higher prices for commercial payers not subject to the negotiation process, which could create situations in which negotiated rebates for nonselected drugs encourage Part D plans to offer a preferred formulary position for nonselected competitors carrying higher prices for patients. While the IRA will cap annual out-of-pocket spending at $2000, most patients do not reach this threshold and thus might be subject to different out-of-pocket costs for preferred vs nonpreferred drugs within a class.

Third, the rules of the exemption aimed at protecting small biotechnology firms may be overly broad and protect some large pharmaceutical firms that were not intended to be covered by the provision. For example, we found that these rules would have prevented Medicare from negotiating prices for extended-release oxycodone and lurasidone, both of which are sold by well-established pharmaceutical companies with billions of dollars in annual revenue. The protections for small biotechnology firms only apply for the first 3 years.

Fourth, the simulation findings highlight the need for CMS to prevent drug manufacturers from avoiding selection by launching new versions of existing products. The IRA instructs CMS to combine spending on different dosage forms before deciding which drugs to select; however, determining which drugs are line extensions and which represent new drugs has historically been difficult for CMS.^23,24^ If Medicare cannot consider multiple versions of a drug together, newer versions may not be eligible for negotiation until years after the original product becomes eligible. For example, we simulated that adalimumab would have been eligible for a negotiated price in 2018. In that same year, the drug’s manufacturer launched a more concentrated, citrate-free version. This new version had greater Medicare spending than the original version by 2020; thus, if CMS only negotiated the price for the original version, this product hop to a new version might have allowed the drug to effectively evade negotiation.

Fifth, estimated savings were high, in part, because ceiling prices erased price increases faster than inflation in the 5 years prior to negotiated prices taking effect. In other words, the ceiling prices for the first negotiated prices set to take effect in 2026 will be based on prices for these drugs in 2021, which may lead to substantial discounts. Some of these savings might be realized by Medicare even without price negotiation because the IRA includes a separate provision requiring manufacturers to pay rebates to offset price increases outpacing inflation. In addition, our simulation was during a period when manufacturers were frequently raising drug prices by 10% or more per year, and inflation was at historically low levels.^25^ The ceiling prices would reflect lower discounts if inflation was higher or if price increases were less pronounced, as might be the case in the years between passage of the IRA and the first negotiated prices taking effect in 2026.

### Limitations

This study has several limitations. First, we simulated the selection of drugs based on the text of the legislation; implementation guidelines and rulemaking from CMS remain in development. Second, we modeled only the first 3 years of negotiation, and savings are likely to grow over time as Medicare selects more drugs and after the ceiling price for drugs approved 12 to 16 years earlier is lowered to 65% of the non-FAMP beginning in 2030. Third, we limited our attention to the drug price negotiation provisions in the IRA and did not examine Part D benefit redesign or inflationary rebates. Fourth, we did not measure savings to Medicare beneficiaries who used drugs with a negotiated price; these savings may be passed on to beneficiaries in the form of lower out-of-pocket costs or premiums. Fifth, we did not address potential savings outside Medicare, which could result from greater bargaining power among private payers to achieve prices similar to the published Medicare negotiated rates.

## Conclusions

In this cross-sectional study using a policy simulation analysis of Medicare drug price negotiation provisions in the IRA from 2018 to 2020, we found that many top-selling drugs did not qualify for negotiation due to strict selection criteria, and a few selected drugs faced generic competition before Medicare had the chance to negotiate prices. Despite these limitations, our findings suggest that the ceiling prices for negotiation would have reduced Medicare prescription drug spending by at least 5% in the first 3 years, and savings may be higher if Medicare can effectively negotiate prices below the statutory ceiling.

## Notes

Supplement 1.
eMethods
**eTable 1.** Top-Selling Drugs Not Eligible Selection, 2016 to 2018 **eTable 2.** Estimated Annual Savings for Simulated Drugs Selected for Negotiation, 2018 to 2020
eReferences

Supplement 2.
Data Sharing Statement
