# Supplementary material for: Simulated Medicare Drug Price Negotiation Under the Inflation Reduction Act of 2022
Source: JAMA Health Forum. 2023 Jan 27;4(1):e225218. doi: 10.1001/jamahealthforum.2022.5218 (PMC11901806; doi:10.1001/jamahealthforum.2022.5218)
Supplement: Supplement 1. — eMethods eTable 1. Top-Selling Drugs Not Eligible Selection, 2016 to 2018 eTable 2. Estimated Annual Savings for Simulated Drugs Selected for Negotiation, 2018 to 2020 eReferences [file jamahealthforum-e225218-s001.pdf]

Supplemental Online Content

Rome BN, Nagar S, Egilman AC, Wang J, Feldman WB, Kesselheim AS. Simulated Medicare drug price negotiation under the Inflation Reduction Act of 2022. *JAMA Health Forum*. 2023;4(1):e225218. doi:10.1001/jamahealthforum.2022.5218

eMethods
eTable 1. Top-Selling Drugs Not Eligible Selection, 2016 to 2018
eTable 2. Estimated Annual Savings for Simulated Drugs Selected for Negotiation, 2018 to 2020
eReferences

This supplemental material has been provided by the authors to give readers additional information about their work.

© 2022 Rome BN et al. *JAMA Health Forum.*

eMethods

Ceiling Prices under the IRA

Under the Inflation Reduction Act (IRA), ceiling prices for negotiation will be based on a drug's non-federal average manufacturer price (non-FAMP). This pricing measure, which is currently used to determine Veterans Affairs administration drug prices, reflects the average price paid to manufacturers by wholesalers, inclusive of discounts and chargebacks but excluding rebates paid by manufacturers to third parties, including payers or pharmacy benefit managers. As such, the non-FAMP is expected to be similar to a drug's list price.

Separate from the negotiation provisions, the IRA includes provisions requiring rebates to offset price increases outpacing inflation since 2021. These protections against price increases are also built into the ceiling price calculations for drugs selected for negotiation. If a drug's price increased faster than inflation since 2021 (5 years prior to the first negotiated prices taking effect in 2026), the ceiling price is based on the non-FAMP from 2021, adjusted for inflationary increases from September 2021 to September of the year prior to selection. In future years, for drugs launched after 2021 the ceiling price will be based on the inflation-adjusted non-FAMP from the year after launch. If a drug's price did not increase faster than inflation since 2021 (or since launch), the non-FAMP from the year prior to selection is used to determine the ceiling price.

© 2022 Rome BN et al. *JAMA Health Forum.*

Estimated savings from ceiling prices

We calculated savings for each drug by multiplying reported pre-rebate Medicare spending by the difference between the current discount percentage and the discount to achieve the ceiling price. For example, if a drug had $1 billion in pre-rebate spending, existing discounts of 20% off the list price, and a ceiling price discount of 35%, the estimated savings would be $150 million ($1 billion x [35% – 20%]). Only 3 top-selling Part D drugs also had spending in Part B (representing <10% of their Part D spending), so we conservatively excluded Part B savings in these cases. Each of the selected top-selling Medicare Part B drugs also had sizeable Part D spending, so in these cases we calculated savings in both programs. We calculated existing discounts from drugs' wholesale acquisition cost (WAC, i.e. list price), but Medicare spending is based on retail prices, which average 7% lower than WAC.[1] We reduced all discounts accordingly to account for this difference.

To estimate existing discounts for Medicare Part D drugs, we used 4-quarter rolling average non-Medicaid rebate estimates from SSR Health. These data may overestimate Medicare discounts for some drugs, particularly those with high statutory discounts in Medicaid and the 340B discount program.[2] However, SSR health provides the most comprehensive drug-specific rebate estimates available, and overestimating existing rebates would conservatively underestimate savings from the IRA. For drugs without SSR Health rebate estimates, we used class-specific rebates based on previously described methods,[2] except for lenalidomide [Revlimid]), which provides maximum discounts of 5% according to a congressional report.[3]

© 2022 Rome BN et al. *JAMA Health Forum.*

eTable 1. Top-Selling Drugs Not Eligible Selection, 2016 to 2018

Not eligible in 2016 (Part D)	Medicare Spending; millions USD [a]	Drug type	Years since FDA approval [b]	Date of first generic or biosimilar competition [c]	Reason not eligible for selection
Ledipasvir/sofosbuvir (Harvoni)	7,031	Small molecule	1.3	Aug-20	Age
Esomeprazole (Nexium)	4,360	Small molecule	14.9	Feb 2015	Generic/biosimilar
Insulin detemir (Levemir)	2,883	Biologic	10.6	N/A	Age
Aripiprazole (Abilify)	2,432	Small molecule	13.2	Apr 2015	Generic/biosimilar
**Not eligible in 2017 (Part D)**					
Ledipasvir/Sofosbuvir (Harvoni)	4,399	Small molecule	2.3	N/A	Age
Insulin Glargine (Lantus)	4,214	Biologic	16.8	Aug 2020	Previously selected
Lenalidomide (Revlimid)	2,661	Small molecule	11.1	N/A	Previously selected
Fluticasone/salmeterol (Advair)	2,511	Small molecule	16.4	Feb 2019	Previously selected
Sitagliptin (Januvia)	2,440	Small molecule	10.3	N/A	Previously selected
Rosuvastatin (Crestor)	2,323	Small molecule	13.5	May 2016	Previously selected
Adalimumab (Humira)	2,198	Biologic	14.1	N/A	Previously selected
Tiotropium (Spiriva)	2,126	Small molecule	13.0	N/A	Previously selected
Pregabalin (Lyrica)	2,099	Small molecule	12.1	Jul 2019	Previously selected
Rivaroxaban (Xarelto)	1,955	Small molecule	5.6	N/A	Age
Apixaban (Eliquis)	1,926	Small molecule	4.1	N/A	Age
Insulin Aspart (Novolog)	1,743	Biologic	16.7	N/A	Previously selected
Ezetimibe (Zetia)	1,451	Small molecule	14.3	Dec 2016	Generic/biosimilar
Glatiramer (Copaxone)	1,434	Small molecule	20.1	Jun 2015	Rare disease
Esomeprazole (Nexium)	1,082	Small molecule	15.9	Feb 2015	Generic/biosimilar
Memantine (Namenda)	1,062	Small molecule	13.3	Feb 2018	Previously selected
Palbociclib (Ibrance)	995	Small molecule	2.0	N/A	Age
Ibrutinib (Imbruvica)	978	Small molecule	3.2	N/A	Age
Sofosbuvir (Sovaldi)	932	Small molecule	3.2	N/A	Age
Dimethyl Fumarate (Tecfidera)	932	Small molecule	3.9	Aug 2020	Age
Enzalutamide (Xtandi)	908	Small molecule	4.4	N/A	Age
Oxycodone (Oxycontin)	882	Small molecule	6.8	N/A	Age
Abiraterone (Zytiga)	823	Small molecule	5.8	Nov 2018	Age
Lurasidone (Latuda)	783	Small molecule	6.3	N/A	Age
Imatinib (Gleevec)	762	Small molecule	15.7	Feb 2016	Generic/biosimilar
Linagliptin (Tradjenta)	708	Small molecule	5.8	N/A	Age
Dabigatran (Pradaxa)	609	Small molecule	6.3	N/A	Age
Canagliflozin (Invokana)	609	Small molecule	3.8	N/A	Age

© 2022 Rome BN et al. *JAMA Health Forum.*

Quetiapine (Seroquel)	560	Small molecule	9.2	Nov 2016	Generic/biosimilar competition
Ruxolitinib (Jakafi)	537	Small molecule	5.2	N/A	Age
**Selected in 2018 (Part B and Part D)**					
Insulin Glargine (Lantus)	4,184	Biologic	17.8	Aug 2020	Previously selected
Lenalidomide (Revlimid)	3,311	Small molecule	12.1	N/A	Previously selected
Apixaban (Eliquis)	3,078	Small molecule	5.1	N/A	Age
Sitagliptin (Januvia)	2,785	Small molecule	11.3	N/A	Previously selected
Adalimumab (Humira)	2,637	Biologic	15.1	N/A	Previously selected
Rivaroxaban (Xarelto)	2,611	Small molecule	6.6	N/A	Age
Fluticasone/salmeterol (Advair)	2,602	Small molecule	17.4	Feb 2019	Previously selected
Ledipasvir/sofosbuvir (Harvoni)	2,552	Small molecule	3.3	N/A	Age
Pregabalin (Lyrica)	2,516	Small molecule	13.1	Jul 2019	Previously selected
Aflibercept (Eylea) [d]	2,479	Biologic	6.2	N/A	Age
Insulin aspart (Novolog)	2,234	Biologic	17.7	N/A	Previously selected
Tiotropium (Spiriva)	2,088	Small molecule	13.0	N/A	Previously selected
Insulin Detemir (Levemir)	1,878	Biologic	15.1	N/A	Previously selected
Insulin Lispro (Humalog)	1,870	Biologic	21.6	N/A	Previously selected
Etanercept (Enbrel)	1,760	Biologic	19.2	N/A	Previously selected
Budesonide/formoterol (Symbicort)	1,510	Small molecule	11.5	N/A	Previously selected
Nivolumab (Opdivo) [d]	1,499	Biologic	2.9	N/A	Age
Glatiramer (Copaxone)	1,499	Small molecule	21.1	Jun 2015	Rare disease
Denosumab (Prolia) [d]	1,479	Biologic	7.7	N/A	Age
Pegfilgrastim (Neulasta) [d]	1,477	Biologic	16.0	Jul 2018	Biosimilar delay
Cinacalcet (Sensipar)	1,436	Small molecule	13.9	Dec 2018	Previously selected
Infliximab (Remicade) [d]	1,424	Biologic	19.4	Dec 2016	Generic/biosimilar
Palbociclib (Ibrance)	1,398	Small molecule	3.0	N/A	Age
Ibrutinib (Imbruvica)	1,368	Small molecule	4.2	N/A	Age
Sevelamer (Renvela)	1,316	Small molecule	10.3	Jun 2017	Previously Selected
Liraglutide (Victoza)	1,295	Small molecule	8.0	N/A	Previously selected
Paliperidone (Invega Sustenna)	1,143	Small molecule	8.5	N/A	Previously selected
Cyclosporine (Restasis)	1,132	Small molecule	14.3	N/A	Previously selected
Bevacizumab (Avastin) [d]	1,091	Biologic	13.9	Jul 2019	Biosimilar delay
Pembrolizumab (Keytruda) [d]	1,048	Biologic	3.4	N/A	Age
Dimethyl (Tecfidera)	1,023	Small molecule	4.9	Aug 2020	Age
Lurasidone (Latuda)	991	Small molecule	7.3	N/A	Small biotech
Sofosbuvir/velpatasvir (Epclusa)	940	Small molecule	1.6	N/A	Age
Abiraterone (Zytiga)	935	Small molecule	6.8	Nov 2018	Age
Memantine (Namenda)	897	Small molecule	14.3	Feb 2018	Previously selected

© 2022 Rome BN et al. *JAMA Health Forum.*

Linagliptin (Tradjenta)	876	Small molecule	6.8	Mar 2023	Age
Enzalutamide (Xtandi)	854	Small molecule	5.4	N/A	Age
Sitagliptin/metformin (Janumet)	835	Small molecule	10.8	N/A	Previously selected
Fluticasone/Vilanterol (Breo Ellipta)	812	Small molecule	4.7	N/A	Age
Trastuzumab (Herceptin) [d]	797	Biologic	19.4	Jul 2019	Biosimilar delay
Mirabegron (Myrbetriq)	787	Small molecule	5.6	N/A	Age
Oxycodone (Oxycontin)	780	Small molecule	7.8	N/A	Small biotech
Ruxolitinib (Jakafi)	724	Small molecule	6.2	N/A	Age
Canagliflozin (Invokana)	717	Small molecule	4.8	N/A	Age
Immune Globulins (Gammagard) [d]	699	Biologic	4.3	N/A	Plasma product
Dulaglutide (Trulicity)	699	Biologic	3.4	N/A	Age
Solifenacin (Vesicare)	697	Small molecule	13.2	Apr 2019	Previously selected
Ezetimibe (Zetia)	696	Small molecule	15.3	Dec 2016	Generic/biosimilar
Abacavir/Dolutegravir/Lamivudine (Triumeq)	651	Small molecule	3.4	N/A	Age
Pomalidomide (Pomalyst)	639	Small molecule	5.0	N/A	Age
Ambrisentan (Letairis)	627	Small molecule	10.6	Apr 2019	Previously selected
Dexlansoprazole (Dexilant)	588	Small molecule	9.0	N/A	Previously selected
Linaclotide (Linzess)	583	Small molecule	5.4	N/A	Age
Teriflunomide (Aubagio)	563	Small molecule	5.4	Nov 2018	Age
Lipase/Protease/Amylase (Creon)	554	Biologic	8.8	N/A	Age
Insulin Glargine (Toujeo)	542	Biologic	2.9	N/A	Age
Elbasvir/grazoprevir (Zepatier)	520	Small molecule	2.0	N/A	Age
Dolutegravir (Tivicay)	518	Small molecule	4.5	N/A	Age
Bimatoprost (Lumigan)	517	Small molecule	16.9	May 2015	Generic/biosimilar
Ipratropium/Albuterol (Combivent)	508	Small molecule	6.3	N/A	Age
Bortezomib (Velcade) [d]	500	Small molecule	14.7	Jan 2018	Generic/biosimilar
Nintedanib (Ofev)	494	Small molecule	3.3	N/A	Age
Pneumococcal vaccine (Prevnar 13) [d]	477	Biologic	6.1	N/A	Age
Pirfenidone (Esbriet)	477	Small molecule	3.3	N/A	Age
Certolizumab Pegol (Cimzia) [d]	470	Biologic	9.8	N/A	Age
Daratumumab (Darzalex)	455	Biologic	2.2	N/A	Age
Esomeprazole (Nexium)	454	Small molecule	16.9	Feb 2015	Generic/biosimilar
Emtricitabine/tenofovir disoproxil fumarate (Truvada)	417	Small molecule	13.5	Sep 2020	Previously selected
Efavirenz/emtricitabine/tenofovir disoproxil fumarate (Atripla)	415	Small molecule	11.6	Oct 2020	Previously selected

[a] Spending is in the full calendar year prior to selection. In 2016 and 2017, spending includes Part D spending only, and in 2018, spending in Part B and D spending were combined.

[b] Age is calculated from the date of FDA approval through February 1 of the selection year (2016, 2017, or 2018).

© 2022 Rome BN et al. *JAMA Health Forum.*

[c] Through December 2020.
[d] Selected from the top-selling drugs in Medicare Part B. All other drugs were top selling drugs in Medicare Part D.

© 2022 Rome BN et al. *JAMA Health Forum.*

eTable 2. Estimated Annual Savings for Simulated Drugs Selected for Negotiation, 2018 to 2020

	**Minimum savings from negotiation** (millions USD) [a]			
**Selected in 2016 (Part D)**	**2018**	**2019**	**2020**	**Total**
Insulin glargine (Lantus)	207	0	0	207
Rosuvastatin (Crestor) [a]	N/a[b]	N/a[b]	N/a[b]	0
Fluticasone/salmeterol (Advair)	68	0	N/a[b]	68
Tiotropium (Spiriva)	330	325	204	859
Sitagliptin (Januvia)	0	0	0	0
Lenalidomide (Revlimid)	2,429	2,943	3,485	8,856
Pregabalin (Lyrica)	1,209	563	88	1,860
Insulin aspart (Novolog)	133	23	-	157
Adalimumab (Humira)	1,496	2,068	2,378	5,942
Memantine (Namenda)	0	N/a[b]	N/a[b]	0
**Selected in 2017 (Part D)**				
Insulin detemir (Levemir)		0	0	0
Insulin lispro (Humalog)		139	59	198
Etanercept (Enbrel)		966	470	1,436
Sevelamer (Renvela)		N/a[b]	N/a[b]	0
Budesonide/formoterol (Symbicort)		0	0	0
Cinacalcet (Sensipar)		42	N/a[b]	42
Liraglutide (Victoza)		274	172	446
Cyclosporine (Restasis)		140	385	525
Paliperidone (Invega Sustenna)		824	981	1,805
Sitagliptin/metformin (Janumet)		0	0	0
Solifenacin (Vesicare)		0	3	3
Emtricitabine/tenofovir (Truvada)		52	13	65
Ambrisentan (Letairis)		230	95	325
Dexlansoprazole (Dexilant)		0	0	0
Efavirenz/emtricitabine/tenofovir (Atripla)		38	21	60
**Selected in 2018 (Part B and Part D)**				
Ranibizumab (Lucentis) [e]			245	245
Abatacept (Orencia) [e]			754	754
Corticotropin (Acthar)			202	202
Dabigatran (Pradaxa)			142	142
Insulin aspart mix (Novolog Mix 70/30)			0	0
Ranolazine (Ranexa)			N/a[b]	0
Teriparatide (Forteo)			168	168
Rifaximin (Xifaxan)			339	339
Omalizumab (Xolair) [e]			421	421
Pemetrexed (Alimta) [e]			230	230
Octreotide (Sandostatin Lar) [e]			389	389
Albuterol (Ventolin)			19	19
Lacosamide (Vimpat)			360	360
Fingolimod (Gilenya)			345	345
Albuterol (Proair)			0	0

[a] If existing discounts (e.g. rebates negotiated by individual Part D plans) exceeded the statutory minimum discount, the ceiling price was set at the average net price; this offered no additional savings to Medicare beyond these existing discounts.

[b] Drug was no longer eligible for a negotiated price due to generic or biosimilar competition.

eReferences

1. Congressional Budget Office. *A Comparison of Brand-Name Drug Prices Among Selected Federal Programs*.; 2021. Accessed June 16, 2021. https://www.cbo.gov/publication/56978

2. Feldman WB, Rome BN, Raimond VC, Gagne JJ, Kesselheim AS. Estimating Rebates and Other Discounts Received by Medicare Part D. *JAMA Health Forum*. 2021;2(6):e210626. doi:10.1001/jamahealthforum.2021.0626

3. Committee on Oversight and Reform. *Drug Pricing Investigation: Celgene and Bristol Myers Squibb—Revlimid*. U.S. House of Representatives; 2020. Accessed August 26, 2022. https://oversight.house.gov/sites/democrats.oversight.house.gov/files/Celgene%20BMS%20Staff%20Report%2009-30-2020.pdf

© 2022 Rome BN et al. *JAMA Health Forum.*
