# Supplementary material for: Simulated Medicare Drug Price Negotiation Under the Inflation Reduction Act of 2022
Source: JAMA Health Forum. 2023 Jan 27;4(1):e225218. doi: 10.1001/jamahealthforum.2022.5218 (PMC11901806; doi:10.1001/jamahealthforum.2022.5218)
Supplement: Supplement 2. — Data Sharing Statement [file jamahealthforum-e225218-s002.pdf]

Data Sharing Statement

Rome. Simulated Medicare Drug Price Negotiation Under the Inflation Reduction Act of 2022. *JAMA Health Forum*. Published January 27, 2023. doi:10.1001/jamahealthforum.2022.5218

Data
Data available: Yes
Data types: Data (not involving human participants)
How to access data: Data can be requested from the corresponding author, Dr. Benjamin Rome (brome@bwh.harvard.edu)
When available: With publication

Supporting Documents
Document types: None

Additional Information
Who can access the data: Anyone requesting the data.
Types of analyses: Any reasonable use as determined by the study authors.
Mechanisms of data availability: With investigator support.
